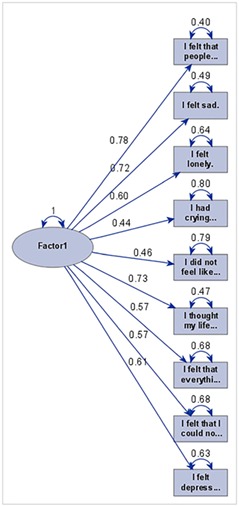# CORRIGENDUM

**DOI:** 10.1002/mpr.1808

**Published:** 2019-12-29

1

Corrigendum to MPR1771 (examining item content validity using property fitting analysis via multidimensional scaling)

“The authors regret an error in Figure 4, two variables are missing from the figure.”

See corrected figure.